# Supplementary material for: Occurrence and Risk Factors of Relapse Activity after Vaccination against COVID-19 in People with Multiple Sclerosis: 1-Year Follow-Up Results from a Nationwide Longitudinal Observational Study
Source: Vaccines (Basel). 2023 Dec 16;11(12):1859. doi: 10.3390/vaccines11121859 (PMC10747540; doi:10.3390/vaccines11121859)
Supplement: Supplementary file 1 [file vaccines-11-01859-s001.zip › REV_Supplementary Figure S1.pdf]

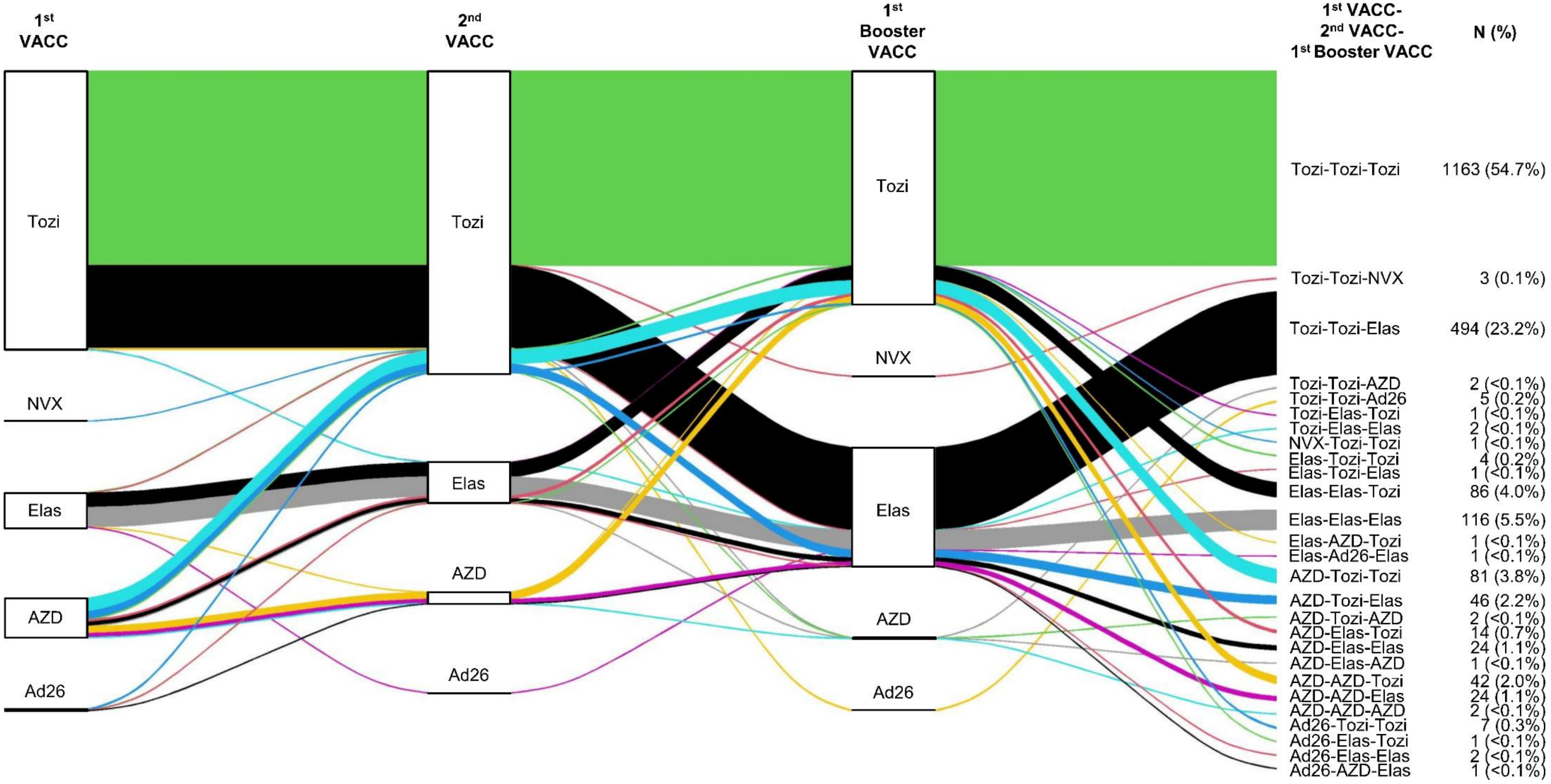

Supplementary Figure S1. Vaccination schedules of the study population. This alluvial graph visualizes the distribution of SARS-CoV-2 vaccines used from the 1st VACC to the 1st booster VACC. Only PwMS who received three VACCs and provided complete VACC data are shown (N=2127). The sizes of the boxes and colored lines symbolize the proportions of PwMS who received the respective vaccine. The most frequent VACC schedule was three administrations of tozinameran (54.7%), followed by the combination of different mRNA-based vaccines (27.6%; tozinameran, elasomeran) and the combination of various mRNA-, vector-based and protein-based vaccines (12.1%; tozinameran, elasomeran, AZD1222, Ad26.COV2.S, NVX-CoV2373). Ad26 – Ad26.COV2.S; AZD – AZD1222; Elas – elasomeran; MS – multiple sclerosis; N – number of patients; NVX – NVX-CoV2373; PwMS – people with MS; Tozi – tozinameran; SARS-CoV-2 – severe acute respiratory syndrome coronavirus 2; VACC – SARS-CoV-2 vaccination.
