# Supplementary material for: Occurrence and Risk Factors of Relapse Activity after Vaccination against COVID-19 in People with Multiple Sclerosis: 1-Year Follow-Up Results from a Nationwide Longitudinal Observational Study
Source: Vaccines (Basel). 2023 Dec 16;11(12):1859. doi: 10.3390/vaccines11121859 (PMC10747540; doi:10.3390/vaccines11121859)
Supplement: Supplementary file 1 [file vaccines-11-01859-s001.zip › REV_Supplementary Figure S2.pdf]

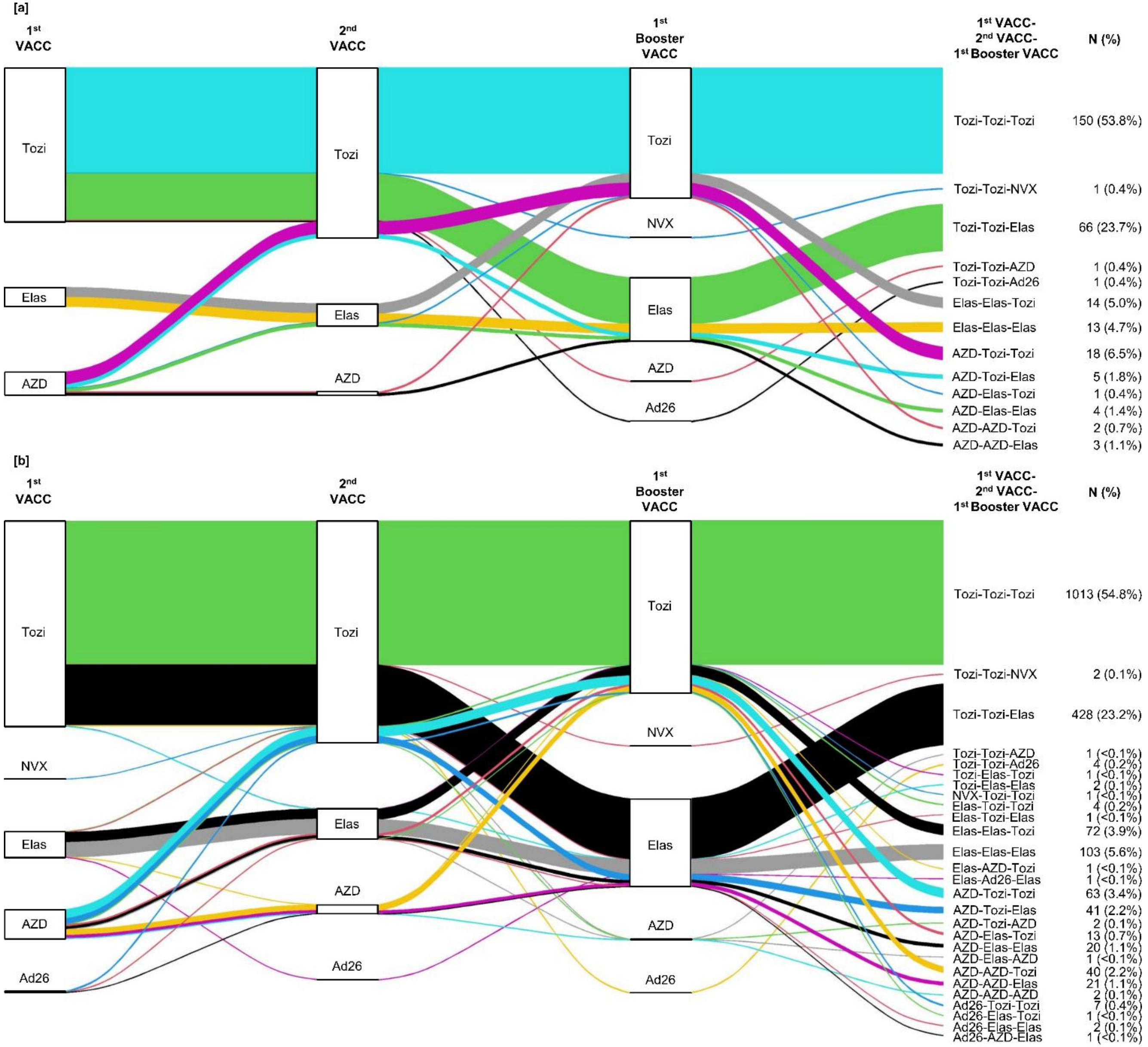

Supplementary Figure S2. Vaccination schedules of people with MS stratified by relapse activity following vaccination against SARS-CoV-2. The distribution of SARS-CoV-2 vaccines used from the 1st VACC to the 1st booster VACC among PwMS with [a] (N=279) and without [b] (N=1848) PV relapses are shown in the alluvial graph. This analysis included only PwMS who received three VACC and provided complete data on VACC. The proportions of PwMS who received the respective vaccine were indicated by the size of the boxes and colored lines. In MS patients with PV relapses, the most common VACC schedule was three administrations of tozinameran (53.8%), followed by the combination of different mRNA-based vaccines (28.7%; tozinameran, elasomeran) and the combination of various mRNA- and vector-based vaccines (12.9%; tozinameran, elasomeran, AZD1222, Ad26.COV2.S). A similar distribution of vaccines was observed in MS patients without PV relapses. Ad26 – Ad26.COV2.S; AZD – AZD1222; Elas – elasomeran; MS – multiple sclerosis; N – number of patients; NVX – NVX-CoV2373; PV – post-vaccination; PwMS – people with MS; Tozi – tozinameran; SARS-CoV-2 – severe acute respiratory syndrome coronavirus 2; VACC – SARS-CoV-2 vaccination.
