# Supplementary material for: Occurrence and Risk Factors of Relapse Activity after Vaccination against COVID-19 in People with Multiple Sclerosis: 1-Year Follow-Up Results from a Nationwide Longitudinal Observational Study
Source: Vaccines (Basel). 2023 Dec 16;11(12):1859. doi: 10.3390/vaccines11121859 (PMC10747540; doi:10.3390/vaccines11121859)
Supplement: Supplementary file 1 [file vaccines-11-01859-s001.zip › REV_Supplementary Figure S3.pdf]

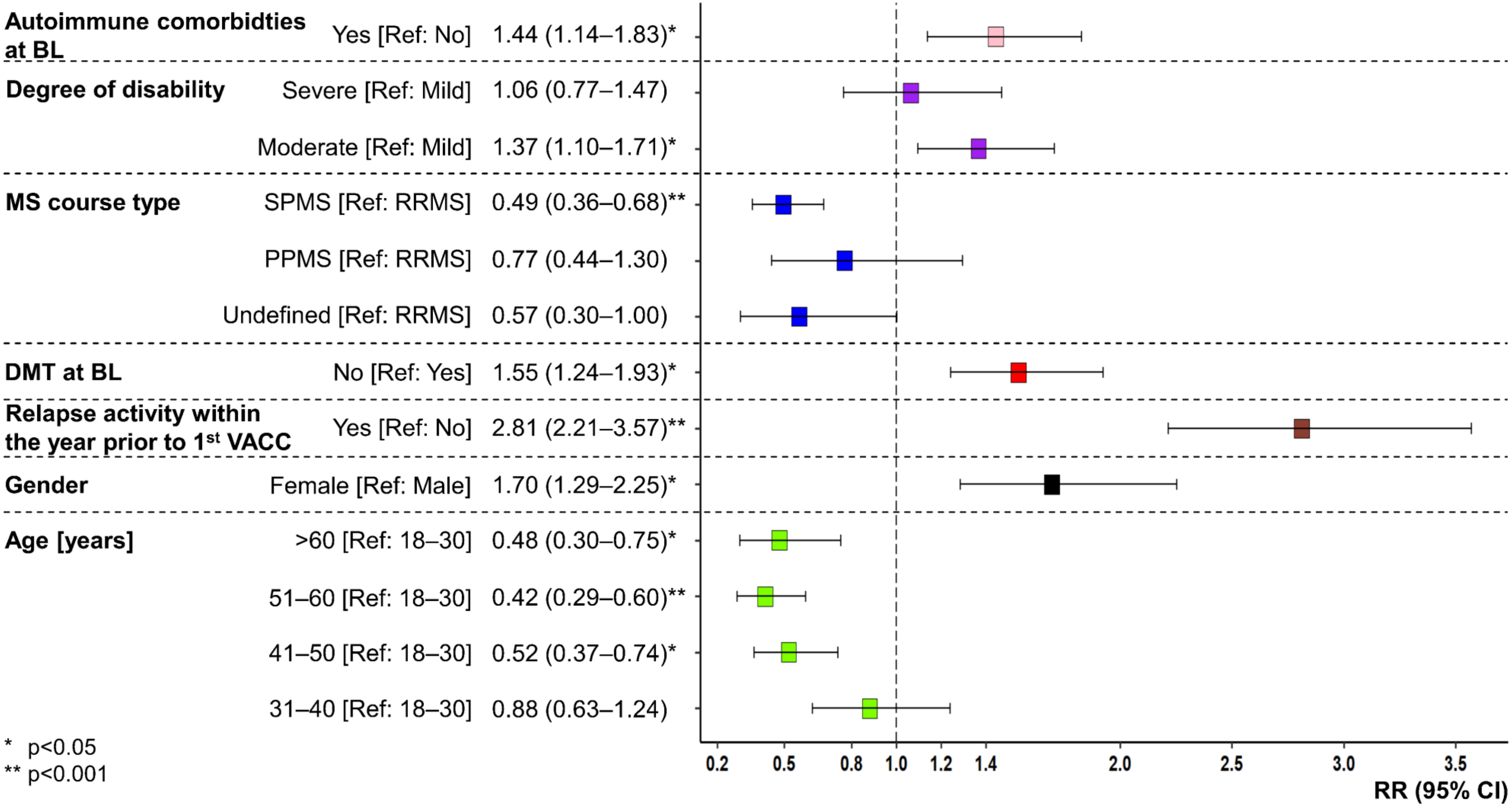

Supplementary Figure S3. Risk factors of post-vaccination relapses in people with MS according to a univariable negative binomial model. RRs (green boxes) and 95% CIs (whiskers) for the occurrence of PV relapses were calculated. Higher risk of PV relapses was found for female gender, moderate disability level, no DMT at baseline, relapses within the year before the first VACC and autoimmune comorbidities. Lower risk of PV relapses was significantly associated with older age and SPMS. BL – baseline; CI – confidence interval; DMT – disease-modifying therapy; MS – multiple sclerosis; p – p-value; PPMS – primary progressive MS; PwMS – people with MS; ref – reference; RR – risk ratio; RRMS – relapsing-remitting MS; SARS-CoV-2 – severe acute respiratory syndrome coronavirus 2; SPMS – secondary progressive MS; VACC – vaccination against SARS-CoV-2.
