# Supplementary material for: Occurrence and Risk Factors of Relapse Activity after Vaccination against COVID-19 in People with Multiple Sclerosis: 1-Year Follow-Up Results from a Nationwide Longitudinal Observational Study
Source: Vaccines (Basel). 2023 Dec 16;11(12):1859. doi: 10.3390/vaccines11121859 (PMC10747540; doi:10.3390/vaccines11121859)
Supplement: Supplementary file 1 [file vaccines-11-01859-s001.zip › REV_Supplementary File S1.pdf]

Supplementary File S1. Third follow up questionnaire

MS Immunotherapy

Have you changed or stopped your MS immunotherapy **<u>since the 1st COVID vaccination</u>**?

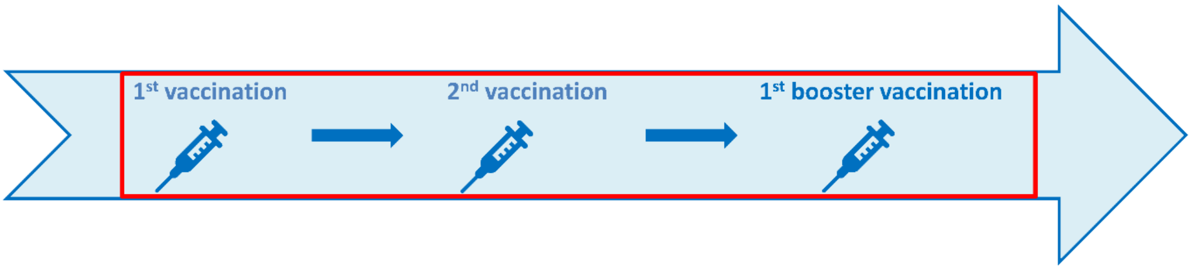

☐ Yes	☐ No
**<u>If yes</u>**, how are you currently being treated?	
☐ Alemtuzumab / Lemtrada	☐ Azathioprine / Imurek
☐ Cladribine / Mavenclad	☐ Cyclophosphamide / Endoxan
☐ Daclizumab / Zinbryta	☐ Dimethyl fumarate / Tecfidera
☐ Fingolimod / Gilenya	☐ Glatiramer acetate / Clift
☐ Glatiramer acetate / Copaxone	☐ Immunglobulin / IVIG / Octagam
☐ Interferon beta-1a / Avonex	☐ Interferon beta-1a / Rebif22
☐ Interferon beta-1a / Rebif44	☐ Interferon beta-1b / Betaferon
☐ Interferon beta-1b / Extavia	☐ Methotrexate
☐ Mitoxantrone / Novatron	☐ Mitoxantrone / Ralenova
☐ Natalizumab / Tysabri	☐ Ocrelizumab / Ocrevus
☐ Ofatumumab / Kesimpta	☐ Ozanimod / Zeposia
☐ Peginterferon beta-1a / Plegridy	☐ Ponesimod / Ponvory
☐ Rituximab / MabThera	☐ Siponimod / Mayzent
☐ Steroids - Long-term therapy, longer than 2 months	☐ Steroids – Pulse therapy
☐ Steroids intrathecal	☐ Teriflunomide / Aubagio
☐ No treated at all (therapy break with planned continuation/switch)	☐ No treated at all (therapy stop without planned continuation/switch)
☐ Other:	
How long have you been treated with the current immunotherapy?	
__.____ (MM.YYYY)	
Have there been any other changes in therapy between the immunotherapy at the time of the 1st COVID vaccination and the current immunotherapy?	
☐ Yes	☐ No
**<u>If yes,</u>** which of the following therapies did you receive during this time (multiple answers possible)?	
☐ Alemtuzumab / Lemtrada	☐ Azathioprine / Imurek
☐ Cladribine / Mavenclad	☐ Cyclophosphamide / Endoxan
☐ Daclizumab / Zinbryta	☐ Dimethyl fumarate / Tecfidera
☐ Fingolimod / Gilenya	☐ Glatiramer acetate / Clift
☐ Glatiramer acetate / Copaxone	☐ Immunglobulin / IVIG / Octagam
☐ Interferon beta-1a / Avonex	☐ Interferon beta-1a / Rebif22
☐ Interferon beta-1a / Rebif44	☐ Interferon beta-1b / Betaferon
☐ Interferon beta-1b / Extavia	☐ Methotrexate
☐ Mitoxantrone / Novatron	☐ Mitoxantrone / Ralenova
☐ Natalizumab / Tysabri	☐ Ocrelizumab / Ocrevus

☐ Ofatumumab / Kesimpta	☐ Ozanimod / Zeposia
☐ Peginterferon beta-1a / Plegridy	☐ Rituximab / MabThera
☐ Siponimod / Mayzent	☐ Steroids - Long-term therapy, longer than 2 months
☐ Steroids – Pulse therapy	☐ Steroids intrathecal
☐ Teriflunomide / Aubagio	☐ Other:
☐ None at all (therapy break/end)	

MS relapses following vaccinations

Have you had any clinically confirmed MS relapses **since the 1st COVID vaccination**?

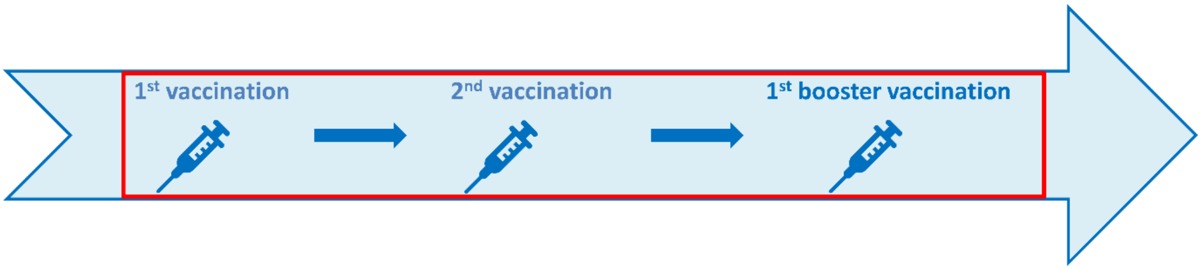

☐ Yes	☐ No

A relapse is characterised by the appearance of new symptoms or the worsening of existing symptoms that last longer than 24 hours. These symptoms can vary greatly and usually develop within a few hours or days. After the relapse, the symptoms may decrease or disappear completely, depending on the course of the disease.

To be able to assume that there is another relapse, there must be at least 30 consecutive days between relapses.

If yes: How many MS relapses occurred and when were they diagnosed?

___ MS relapse/s since the 1st vaccination until the 1st booster vaccination (**for basic immunization with one vaccination**)
The 1st relapse since the 1st vaccination until the 1st booster vaccination was diagnosed on _________ **(DD.MM.YYYY)**.
The Xth relapse since the 1st vaccination until the 1st booster vaccination was diagnosed on _________ **(DD.MM.YYYY)**.

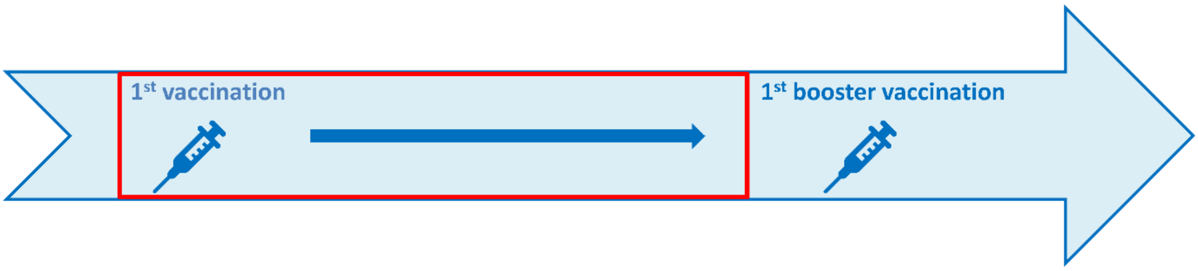

___ MS relapse/s since the 1st until the 2nd vaccination (**for basic immunization with two vaccinations**)
The 1st relapse since the 1st until the 2nd vaccination was diagnosed on _________ **(DD.MM.YYYY)**.

The Xth relapse since the 1st until the 2nd vaccination was diagnosed on _________ **(DD.MM.YYYY)**.

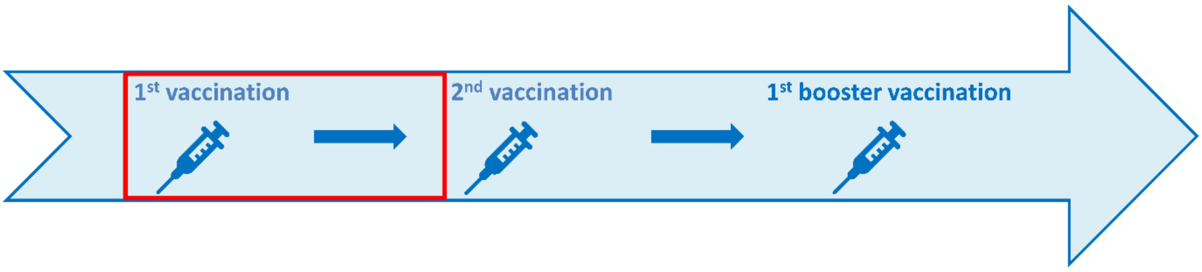

___ MS relapse/s since the 2nd vaccination until the 1st booster vaccination (**for basic immunization with two vaccinations**)

The 1st relapse since the 2nd vaccination until the 1st booster vaccination was diagnosed on _________ **(DD.MM.YYYY)**.

The Xth relapse since the 2nd vaccination until the 1st booster vaccination was diagnosed on _________ **(DD.MM.YYYY)**.

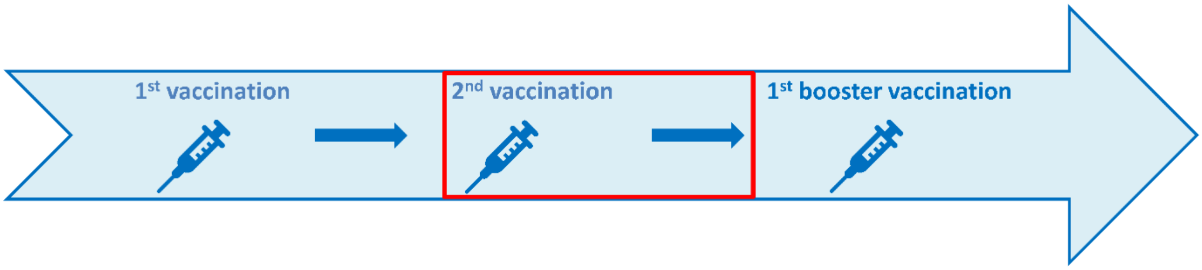

___ MS relapse/s since the 1st booster vaccination (**if booster vaccination received**)

The 1st relapse since the 1st booster vaccination was diagnosed on _________ **(DD.MM.YYYY)**.

The Xth relapse since the 1st booster vaccination was diagnosed on _________ **(DD.MM.YYYY)**.

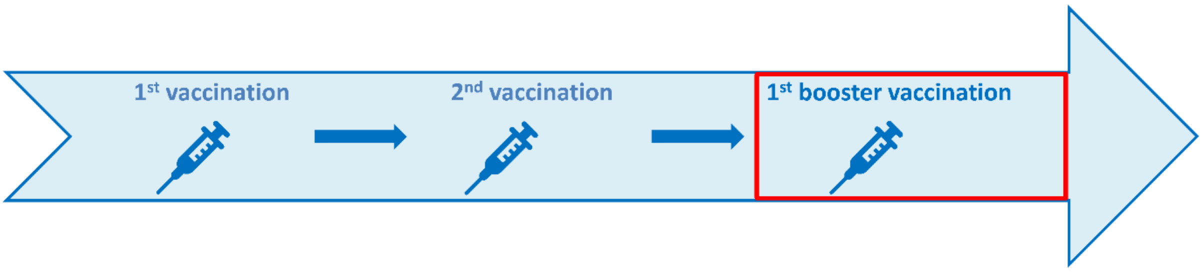

What MS symptoms have you experienced during the relapses?

Please select the information below that applies to you (multiple selections possible).

	Aufgetretene Symptome
Walking impairment	☐
Spasticity	☐
Movement disorders/ataxia/tremor	☐
Tiredness / fatigue	☐
Pain	☐
Bladder dysfunction/impaired micturition	☐
Bowel dysfunction/impaired defecation	☐
Sexual disorders	☐
Cognitive disorders	☐
Depression	☐
Eye movement disorders/oculomotor disorders	☐

Speaking and voice disorders/dysarthria/dysphonia	☐
Swallowing disorders/dysphagia	☐
Epileptic seizures	☐
Palsy/paresis	☐
Optic nerve inflammation/optic neuritis	☐
Other seizure-like symptoms/paroxysms	☐
How many of these relapses were treated with steroid pulse therapy?	
___ relapse/s	

New-onset or worsened MS symptoms

In the following, only symptoms or changes in symptoms that are clearly <u>not</u> related to SARS-CoV-2 infection are asked about because they occurred <u>before</u> this infection.

New-onset MS symptoms

Did you experience new MS symptoms related to the **2nd/last vaccination (excluding booster vaccinations)**?

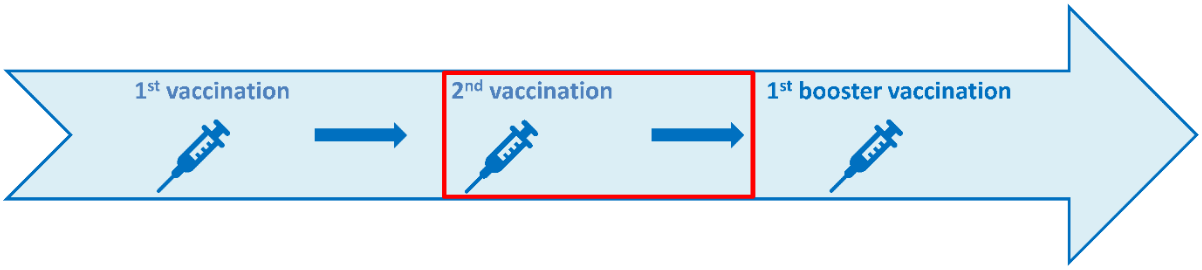

☐ Yes		☐ No
Please select the information below that applies to you.		
**Walking impairment**		
When did it start: _______ **(MM.YYYY)**		
☐ When did it end: after ___ days OR after ___ weeks (**fill in only one of the two**)		☐ Still persistent
**Spasticity**	☐ Yes	☐ No
When did it start: _______ **(MM.YYYY)**		
☐ When did it end: after ___ days OR after ___ weeks (**fill in only one of the two**)		☐ Still persistent
**Movement disorders / ataxia / tremor**	☐ Yes	☐ No
When did it start: _______ **(MM.YYYY)**		
☐ When did it end: after ___ days OR after ___ weeks (**fill in only one of the two**)		☐ Still persistent
**Tiredness / fatigue**	☐ Yes	☐ No
When did it start: _______ **(MM.YYYY)**		
☐ When did it end: after ___ days OR after ___ weeks (**fill in only one of the two**)		☐ Still persistent
**Pain**	☐ Yes	☐ No
When did it start: _______ **(MM.YYYY)**		
☐ When did it end: after ___ days OR after ___ weeks (**fill in only one of the two**)		☐ Still persistent

**Bladder dysfunction / impaired micturition**	☐ Yes	☐ No
When did it start: _______ **(MM.YYYY)**		
☐ When did it end: after ___ days OR after ___ weeks (**fill in only one of the two**)		☐ Still persistent
**Bowel dysfunction / impaired defecation**	☐ Yes	☐ No
When did it start: _______ **(MM.YYYY)**		
☐ When did it end: after ___ days OR after ___ weeks (**fill in only one of the two**)		☐ Still persistent
**Sexual disorders**	☐ Yes	☐ No
When did it start: _______ **(MM.YYYY)**		
☐ When did it end: after ___ days OR after ___ weeks (**fill in only one of the two**)		☐ Still persistent
**Cognitive disorders**	☐ Yes	☐ No
When did it start: _______ **(MM.YYYY)**		
☐ When did it end: after ___ days OR after ___ weeks (**fill in only one of the two**)		☐ Still persistent
**Depression**	☐ Yes	☐ No
When did it start: _______ **(MM.YYYY)**		
☐ When did it end: after ___ days OR after ___ weeks (**fill in only one of the two**)		☐ Still persistent
**Eye movement disorders / oculomotor disorders**	☐ Yes	☐ No
When did it start: _______ **(MM.YYYY)**		
☐ When did it end: after ___ days OR after ___ weeks (**fill in only one of the two**)		☐ Still persistent
**Speaking and voice disorders / dysarthria / dysphonia**	☐ Yes	☐ No
When did it start: _______ **(MM.YYYY)**		
☐ When did it end: after ___ days OR after ___ weeks (**fill in only one of the two**)		☐ Still persistent
**Swallowing disorders / dysphagia**	☐ Yes	☐ No
When did it start: _______ **(MM.YYYY)**		
☐ When did it end: after ___ days OR after ___ weeks (**fill in only one of the two**)		☐ Still persistent
**Epileptic seizures**	☐ Yes	☐ No
When did it start: _______ **(MM.YYYY)**		
☐ When did it end: after ___ days OR after ___ weeks (**fill in only one of the two**)		☐ Still persistent
**Palsy / paresis**	☐ Yes	☐ No
When did it start: _______ **(MM.YYYY)**		
☐ When did it end: after ___ days OR after ___ weeks (**fill in only one of the two**)		☐ Still persistent
**Optic nerve inflammation / optic neuritis**	☐ Yes	☐ No
When did it start: _______ **(MM.YYYY)**		
☐ When did it end: after ___ days OR after ___ weeks (**fill in only one of the two**)		☐ Still persistent

**Other seizure-like symptoms / paroxysms**	☐ Yes	☐ No
When did it start: _______ **(MM.YYYY)**		
☐ When did it end: after ___ days OR after ___ weeks (**fill in only one of the two**)		☐ Still persistent

Worsened MS symptoms

Did you experience worsened MS symptoms related to the **<u>2nd/last vaccination (excluding booster vaccinations)</u>**?

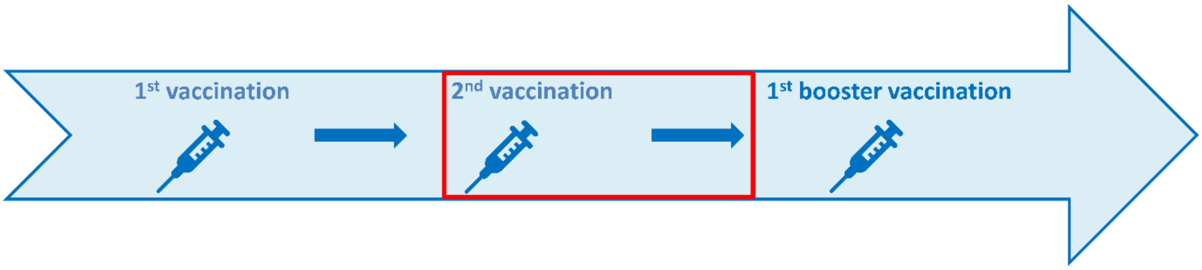

☐ Yes		☐ No
Please select the information below that applies to you.		
**Walking impairment**		
When did it start: _______ **(MM.YYYY)**		
☐ When did it end: after ___ days OR after ___ weeks (**fill in only one of the two**)		☐ Still persistent
**Spasticity**	☐ Yes	☐ No
When did it start: _______ **(MM.YYYY)**		
☐ When did it end: after ___ days OR after ___ weeks (**fill in only one of the two**)		☐ Still persistent
**Movement disorders / ataxia / tremor**	☐ Yes	☐ No
When did it start: _______ **(MM.YYYY)**		
☐ When did it end: after ___ days OR after ___ weeks (**fill in only one of the two**)		☐ Still persistent
**Tiredness / fatigue**	☐ Yes	☐ No
When did it start: _______ **(MM.YYYY)**		
☐ When did it end: after ___ days OR after ___ weeks (**fill in only one of the two**)		☐ Still persistent
**Pain**	☐ Yes	☐ No
When did it start: _______ **(MM.YYYY)**		
☐ When did it end: after ___ days OR after ___ weeks (**fill in only one of the two**)		☐ Still persistent
**Bladder dysfunction / impaired micturition**	☐ Yes	☐ No
When did it start: _______ **(MM.YYYY)**		
☐ When did it end: after ___ days OR after ___ weeks (**fill in only one of the two**)		☐ Still persistent
**Bowel dysfunction / impaired defecation**	☐ Yes	☐ No
When did it start: _______ **(MM.YYYY)**		
☐ When did it end: after ___ days OR after ___ weeks (**fill in only one of the two**)		☐ Still persistent
**Sexual disorders**	☐ Yes	☐ No

When did it start: _______ **(MM.YYYY)**		
☐ When did it end: after ___ days OR after ___ weeks (**fill in only one of the two**)		☐ Still persistent
**Cognitive disorders**	☐ Yes	☐ No
When did it start: _______ **(MM.YYYY)**		
☐ When did it end: after ___ days OR after ___ weeks (**fill in only one of the two**)		☐ Still persistent
**Depression**	☐ Yes	☐ No
When did it start: _______ **(MM.YYYY)**		
☐ When did it end: after ___ days OR after ___ weeks (**fill in only one of the two**)		☐ Still persistent
**Eye movement disorders / oculomotor disorders**	☐ Yes	☐ No
When did it start: _______ **(MM.YYYY)**		
☐ When did it end: after ___ days OR after ___ weeks (**fill in only one of the two**)		☐ Still persistent
**Speaking and voice disorders / dysarthria / dysphonia**	☐ Yes	☐ No
When did it start: _______ **(MM.YYYY)**		
☐ When did it end: after ___ days OR after ___ weeks (**fill in only one of the two**)		☐ Still persistent
**Swallowing disorders / dysphagia**	☐ Yes	☐ No
When did it start: _______ **(MM.YYYY)**		
☐ When did it end: after ___ days OR after ___ weeks (**fill in only one of the two**)		☐ Still persistent
**Epileptic seizures**	☐ Yes	☐ No
When did it start: _______ **(MM.YYYY)**		
☐ When did it end: after ___ days OR after ___ weeks (**fill in only one of the two**)		☐ Still persistent
**Palsy / paresis**	☐ Yes	☐ No
When did it start: _______ **(MM.YYYY)**		
☐ When did it end: after ___ days OR after ___ weeks (**fill in only one of the two**)		☐ Still persistent
**Optic nerve inflammation / optic neuritis**	☐ Yes	☐ No
When did it start: _______ **(MM.YYYY)**		
☐ When did it end: after ___ days OR after ___ weeks (**fill in only one of the two**)		☐ Still persistent
**Other seizure-like symptoms / paroxysms**	☐ Yes	☐ No
When did it start: _______ **(MM.YYYY)**		
☐ When did it end: after ___ days OR after ___ weeks (**fill in only one of the two**)		☐ Still persistent

SARS-CoV-2 infections following vaccinations

How many confirmed SARS-CoV-2 infections have you had **since your 1st COVID vaccination**?

___ confirmed infection/s since the 1st vaccination until the 1st booster vaccination (**for basic immunization with one vaccination**)

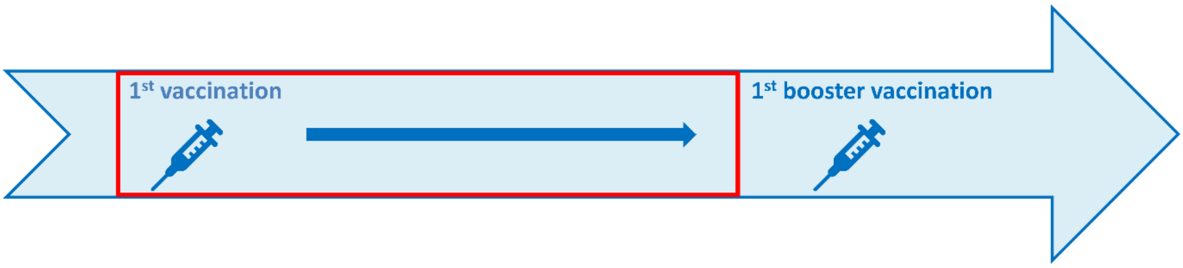

___ confirmed infection/s since the 1st until the 2nd vaccination (**for basic immunization with two vaccinations**)

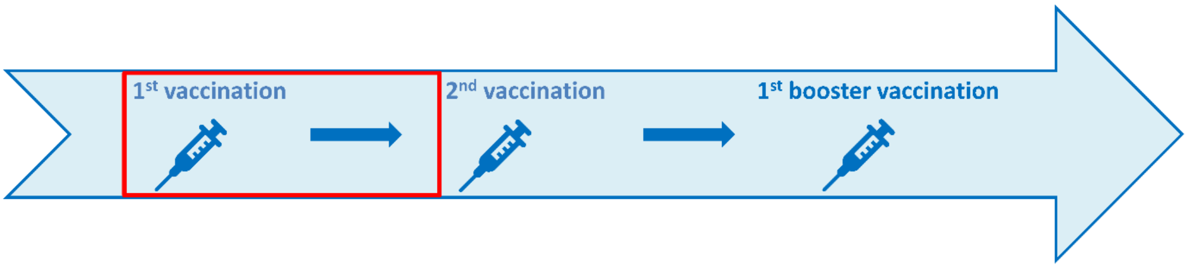

___ confirmed infection/s since the 2nd vaccination until the 1st booster vaccination (**for basic immunization with two vaccinations**)

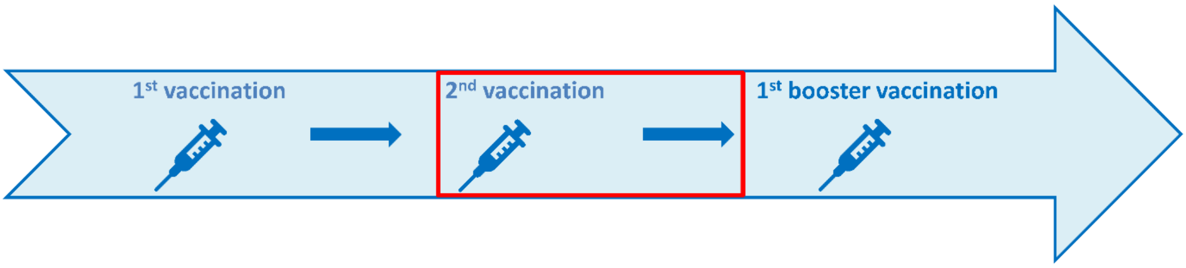

___ confirmed infection/s since the 1st booster vaccination (**if booster vaccination received**)

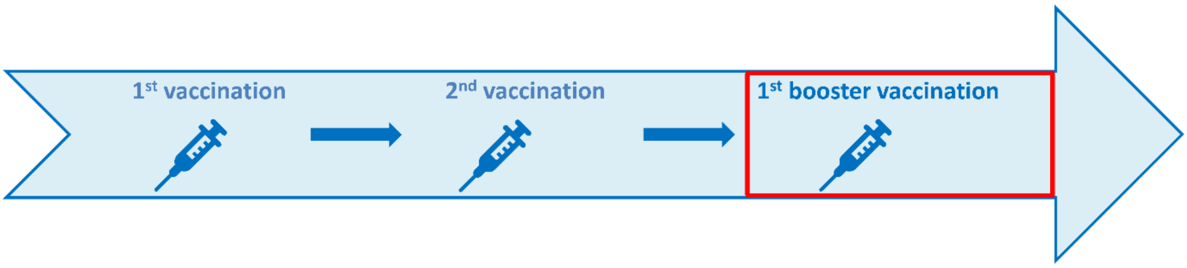

The following questions refer to the 1st SARS-CoV-2 infection **since the 1st until the 2nd vaccination** (**for basic immunization with two vaccinations**)

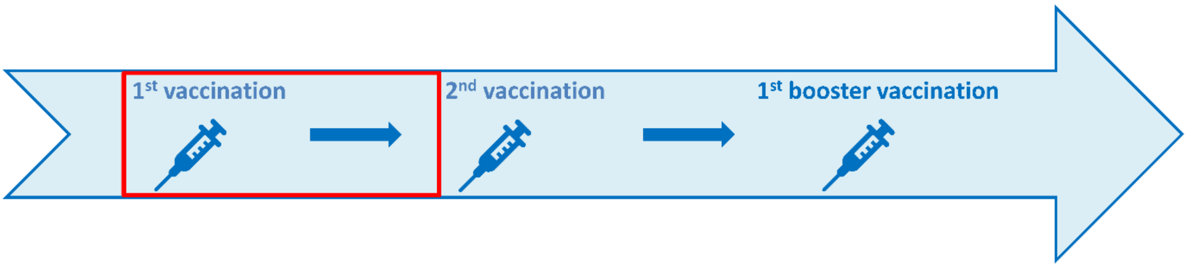

What test was used to detect the infection?

☐ PCR test	☐ Antigen test
☐ Antibody test	☐ ELISPOT test

☐ Unknown	
Please indicate the onset of SARS-CoV-2 infection:	__.____ (MM.YYYY)
What symptoms did you experience?	
☐ Increased temperature	☐ Cough
☐ Lassitude	☐ Joint, bone or muscle pain
☐ Headache	☐ Sore throat
☐ Shortness of breath	☐ Blocked nose
☐ Chills	☐ Loss of smell or taste
☐ Pneumonia	☐ Other: ________________________
For the treatment you were:	
☐ Admitted as an inpatient in a hospital	
☐ Admitted to hospital as an inpatient and treated in intensive care for at least one day.	
☐ Treated at home.	
If they have been admitted to a hospital:	
How many days were you admitted to hospital?	________________
Were you connected to a (ventilated) respirator or did you receive oxygen therapy during your inpatient stay?	
○ Non-invasive treatment	○ Invasive treatment
<u>Non-invasive ventilation:</u> respiratory support or ventilation without invasive ventilation access, for example via a mask placed over the mouth and nose.<br><u>Invasive ventilation:</u> Placement of a breathing tube inserted through the mouth or nose to serve as an artificial airway.	
How many days were you ventilated?	____________________

The following questions refer to the Xth SARS-CoV-2 infection **<u>since the 1st until the 2nd vaccination</u>** (**for basic immunization with two vaccinations**)

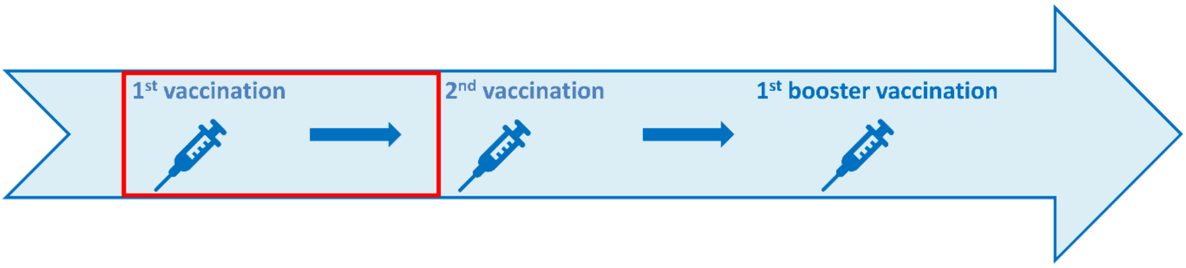

What test was used to detect the infection?	
☐ PCR test	☐ Antigen test
☐ Antibody test	☐ ELISPOT test
☐ Unknown	
Please indicate the onset of SARS-CoV-2 infection:	__.____ (MM.YYYY)
What symptoms did you experience?	
☐ Increased temperature	☐ Cough
☐ Lassitude	☐ Joint, bone or muscle pain
☐ Headache	☐ Sore throat
☐ Shortness of breath	☐ Blocked nose
☐ Chills	☐ Loss of smell or taste
☐ Pneumonia	☐ Other: ________________________

For the treatment you were:	
☐ Admitted as an inpatient in a hospital	
☐ Admitted to hospital as an inpatient and treated in intensive care for at least one day.	
☐ Treated at home.	
If they have been admitted to a hospital:	
How many days were you admitted to hospital?	___________________
Were you connected to a (ventilated) respirator or did you receive oxygen therapy during your inpatient stay?	
○ Non-invasive treatment	○ Invasive treatment
<u>Non-invasive ventilation:</u> respiratory support or ventilation without invasive ventilation access, for example via a mask placed over the mouth and nose.<br><u>Invasive ventilation:</u> Placement of a breathing tube inserted through the mouth or nose to serve as an artificial airway.	
How many days were you ventilated?	______________________

The following questions refer to the 1st SARS-CoV-2 infection **<u>since the 2nd vaccination until the 1st booster vaccination</u>** (**for basic immunization with two vaccinations**)

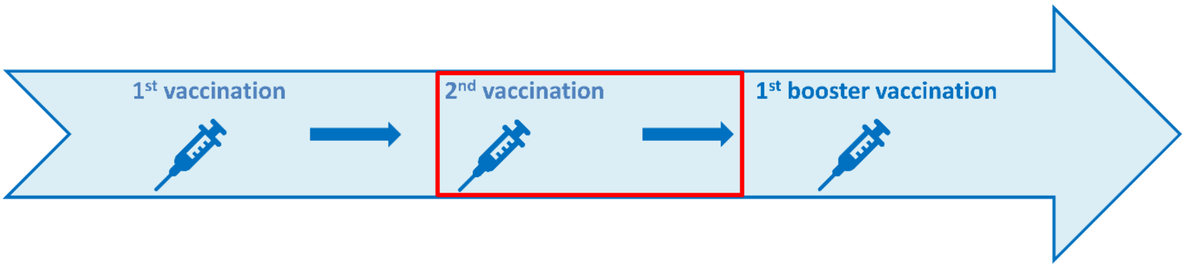

What test was used to detect the infection?	
☐ PCR test	☐ Antigen test
☐ Antibody test	☐ ELISPOT test
☐ Unknown	
Please indicate the onset of SARS-CoV-2 infection:	__.____ (MM.YYYY)
What symptoms did you experience?	
☐ Increased temperature	☐ Cough
☐ Lassitude	☐ Joint, bone or muscle pain
☐ Headache	☐ Sore throat
☐ Shortness of breath	☐ Blocked nose
☐ Chills	☐ Loss of smell or taste
☐ Pneumonia	☐ Other: __________________________
For the treatment you were:	
☐ Admitted as an inpatient in a hospital	
☐ Admitted to hospital as an inpatient and treated in intensive care for at least one day.	
☐ Treated at home.	
If they have been admitted to a hospital:	
How many days were you admitted to hospital?	___________________
Were you connected to a (ventilated) respirator or did you receive oxygen therapy during your inpatient stay?	
○ Non-invasive treatment	○ Invasive treatment

Non-invasive ventilation: respiratory support or ventilation without invasive ventilation access, for example via a mask placed over the mouth and nose.<br>Invasive ventilation: Placement of a breathing tube inserted through the mouth or nose to serve as an artificial airway.

How many days were you ventilated?	____________________

The following questions refer to the Xth SARS-CoV-2 infection **since the 2nd vaccination until the 1st booster vaccination** (**for basic immunization with two vaccinations**)

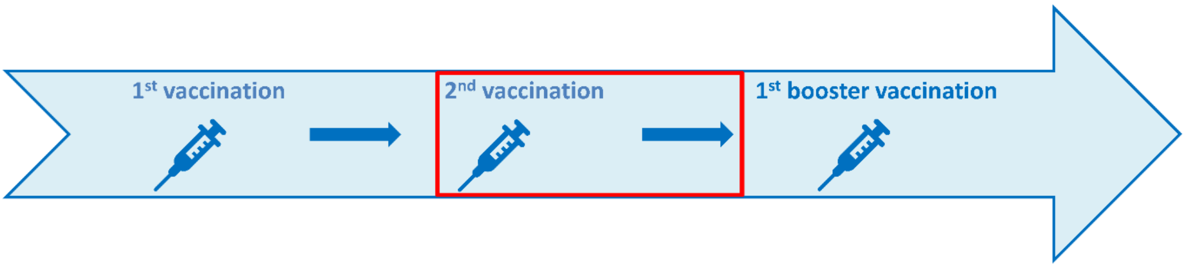

What test was used to detect the infection?	
☐ PCR test	☐ Antigen test
☐ Antibody test	☐ ELISPOT test
☐ Unknown	
Please indicate the onset of SARS-CoV-2 infection:	__.____ (MM.YYYY)
What symptoms did you experience?	
☐ Increased temperature	☐ Cough
☐ Lassitude	☐ Joint, bone or muscle pain
☐ Headache	☐ Sore throat
☐ Shortness of breath	☐ Blocked nose
☐ Chills	☐ Loss of smell or taste
☐ Pneumonia	☐ Other: ______________________
For the treatment you were:	
☐ Admitted as an inpatient in a hospital	
☐ Admitted to hospital as an inpatient and treated in intensive care for at least one day.	
☐ Treated at home.	
If they have been admitted to a hospital:	
How many days were you admitted to hospital?	________________
Were you connected to a (ventilated) respirator or did you receive oxygen therapy during your inpatient stay?	
○ Non-invasive treatment	○ Invasive treatment
Non-invasive ventilation: respiratory support or ventilation without invasive ventilation access, for example via a mask placed over the mouth and nose.<br>Invasive ventilation: Placement of a breathing tube inserted through the mouth or nose to serve as an artificial airway.	
How many days were you ventilated?	____________________

The following questions refer to the 1st SARS-CoV-2 infection **since the 1st vaccination until the 1st booster vaccination** (**for basic immunization with one vaccination**)

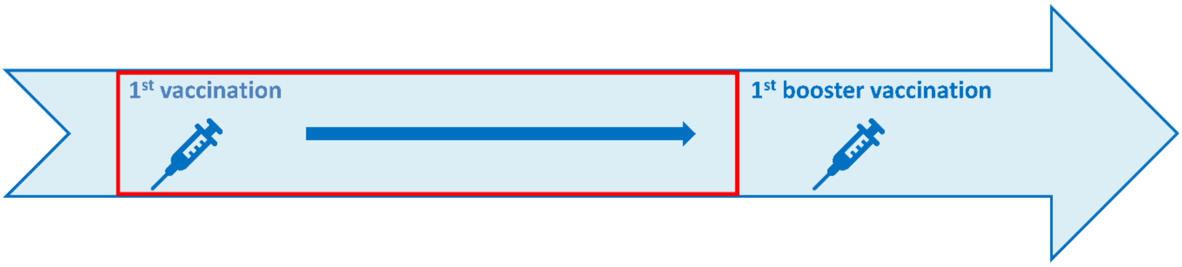

What test was used to detect the infection?	
☐ PCR test	☐ Antigen test
☐ Antibody test	☐ ELISPOT test
☐ Unknown	
Please indicate the onset of SARS-CoV-2 infection:	__.____ (MM.YYYY)
What symptoms did you experience?	
☐ Increased temperature	☐ Cough
☐ Lassitude	☐ Joint, bone or muscle pain
☐ Headache	☐ Sore throat
☐ Shortness of breath	☐ Blocked nose
☐ Chills	☐ Loss of smell or taste
☐ Pneumonia	☐ Other: ____________________________
For the treatment you were:	
☐ Admitted as an inpatient in a hospital	
☐ Admitted to hospital as an inpatient and treated in intensive care for at least one day.	
☐ Treated at home.	
If they have been admitted to a hospital:	
How many days were you admitted to hospital?	____________________
Were you connected to a (ventilated) respirator or did you receive oxygen therapy during your inpatient stay?	
○ Non-invasive treatment	○ Invasive treatment
<u>Non-invasive ventilation:</u> respiratory support or ventilation without invasive ventilation access, for example via a mask placed over the mouth and nose.<br><u>Invasive ventilation:</u> Placement of a breathing tube inserted through the mouth or nose to serve as an artificial airway.	
How many days were you ventilated?	________________________

The following questions refer to the Xth SARS-CoV-2 infection **<u>since the 1st vaccination until the 1st booster vaccination</u>** (**for basic immunization with one vaccination**)

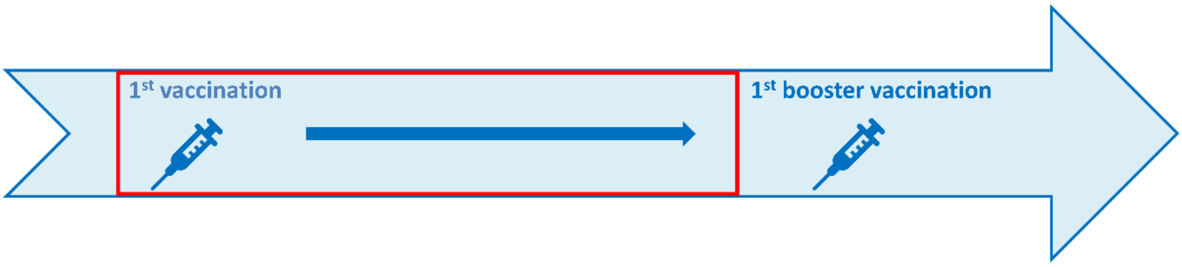

What test was used to detect the infection?	
☐ PCR test	☐ Antigen test
☐ Antibody test	☐ ELISPOT test

☐ Unknown	
Please indicate the onset of SARS-CoV-2 infection:	__.____ (MM.YYYY)
What symptoms did you experience?	
☐ Increased temperature	☐ Cough
☐ Lassitude	☐ Joint, bone or muscle pain
☐ Headache	☐ Sore throat
☐ Shortness of breath	☐ Blocked nose
☐ Chills	☐ Loss of smell or taste
☐ Pneumonia	☐ Other: ______________________
For the treatment you were:	
☐ Admitted as an inpatient in a hospital	
☐ Admitted to hospital as an inpatient and treated in intensive care for at least one day.	
☐ Treated at home.	
If they have been admitted to a hospital:	
How many days were you admitted to hospital?	________________
Were you connected to a (ventilated) respirator or did you receive oxygen therapy during your inpatient stay?	
○ Non-invasive treatment	○ Invasive treatment
<u>Non-invasive ventilation:</u> respiratory support or ventilation without invasive ventilation access, for example via a mask placed over the mouth and nose.<br><u>Invasive ventilation:</u> Placement of a breathing tube inserted through the mouth or nose to serve as an artificial airway.	
How many days were you ventilated?	___________________

The following questions refer to the 1st SARS-CoV-2 infection **<u>since the 1st booster vaccination</u>** (**if booster vaccination received**)	

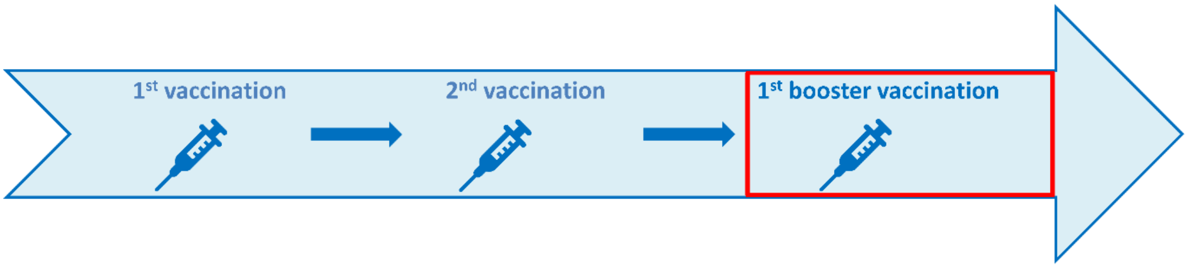

What test was used to detect the infection?	
☐ PCR test	☐ Antigen test
☐ Antibody test	☐ ELISPOT test
☐ Unknown	
Please indicate the onset of SARS-CoV-2 infection:	__.____ (MM.YYYY)
What symptoms did you experience?	
☐ Increased temperature	☐ Cough
☐ Lassitude	☐ Joint, bone or muscle pain
☐ Headache	☐ Sore throat
☐ Shortness of breath	☐ Blocked nose
☐ Chills	☐ Loss of smell or taste
☐ Pneumonia	☐ Other: ______________________

For the treatment you were:	
☐ Admitted as an inpatient in a hospital	
☐ Admitted to hospital as an inpatient and treated in intensive care for at least one day.	
☐ Treated at home.	
If they have been admitted to a hospital:	
How many days were you admitted to hospital?	____________________
Were you connected to a (ventilated) respirator or did you receive oxygen therapy during your inpatient stay?	
○ Non-invasive treatment	○ Invasive treatment
Non-invasive ventilation: respiratory support or ventilation without invasive ventilation access, for example via a mask placed over the mouth and nose.<br>Invasive ventilation: Placement of a breathing tube inserted through the mouth or nose to serve as an artificial airway.	
How many days were you ventilated?	________________________

The following questions refer to the Xth SARS-CoV-2 infection **<u>since the 1st booster vaccination</u>** (**if booster vaccination received**)

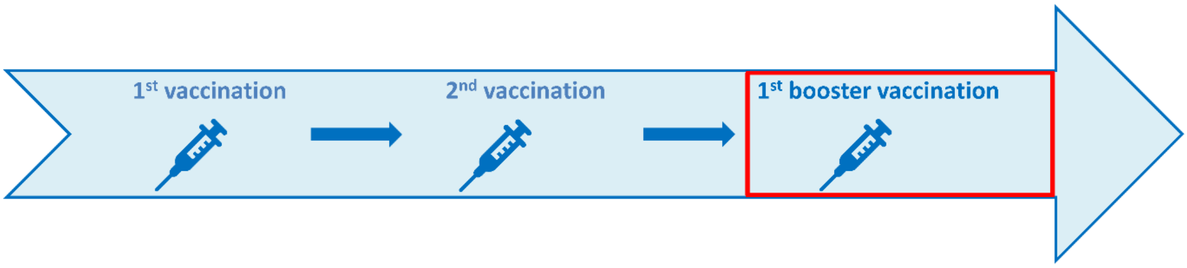

What test was used to detect the infection?	
☐ PCR test	☐ Antigen test
☐ Antibody test	☐ ELISPOT test
☐ Unknown	
Please indicate the onset of SARS-CoV-2 infection:	__.____ (MM.YYYY)
What symptoms did you experience?	
☐ Increased temperature	☐ Cough
☐ Lassitude	☐ Joint, bone or muscle pain
☐ Headache	☐ Sore throat
☐ Shortness of breath	☐ Blocked nose
☐ Chills	☐ Loss of smell or taste
☐ Pneumonia	☐ Other: ____________________________
For the treatment you were:	
☐ Admitted as an inpatient in a hospital	
☐ Admitted to hospital as an inpatient and treated in intensive care for at least one day.	
☐ Treated at home.	
If they have been admitted to a hospital:	
How many days were you admitted to hospital?	____________________
Were you connected to a (ventilated) respirator or did you receive oxygen therapy during your inpatient stay?	
○ Non-invasive treatment	○ Invasive treatment

Non-invasive ventilation: respiratory support or ventilation without invasive ventilation access, for example via a mask placed over the mouth and nose.<br>Invasive ventilation: Placement of a breathing tube inserted through the mouth or nose to serve as an artificial airway.	
How many days were you ventilated?	______________________

After at least one of your SARS-CoV-2 infections, did you suffer from concomitant symptoms for at least 4 weeks (Long Covid)?	☐ Yes	☐ No
If so, what are the symptoms?		
☐ Increased temperature	☐ Cough	
☐ Lassitude	☐ Joint, bone or muscle pain	
☐ Headache	☐ Sore throat	
☐ Shortness of breath	☐ Blocked nose	
☐ Chills	☐ Loss of smell or taste	
☐ Pneumonia	☐ Other: _________________________	
After at least one of your SARS-CoV-2 infections, did you suffer from concomitant symptoms for at least 12 weeks (Post Covid)?	☐ Yes	☐ No
If so, what are the symptoms?		
☐ Increased temperature	☐ Cough	
☐ Lassitude	☐ Joint, bone or muscle pain	
☐ Headache	☐ Sore throat	
☐ Shortness of breath	☐ Blocked nose	
☐ Chills	☐ Loss of smell or taste	
☐ Pneumonia	☐ Other: _________________________	
Are you still experiencing any side effects from your SARS-CoV-2 infection(s) at the time of this survey?	☐ Yes	☐ No
If so, what are the symptoms?		
☐ Increased temperature	☐ Cough	
☐ Lassitude	☐ Joint, bone or muscle pain	
☐ Headache	☐ Sore throat	
☐ Shortness of breath	☐ Blocked nose	
☐ Chills	☐ Loss of smell or taste	
☐ Pneumonia	☐ Other: _________________________	

Tests for immune response after the SARS-CoV-2 vaccinations

Antibody test:

Have you had an antibody test **since the 1st until the 2nd vaccination** (**for basic immunization with two vaccinations**)?

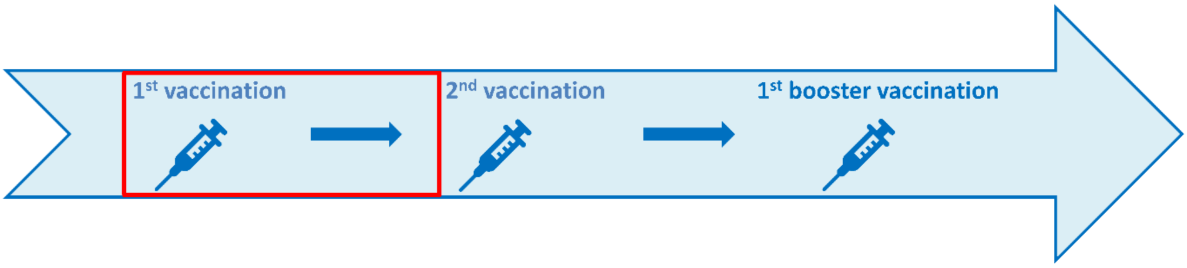

☐ Yes, antibodies positive

☐ Yes, antibodies negative

☐ No

Date of the (last) antibody test (DD.MM.YYYY): ________

Below you will find two examples of antibody test results. Please fill in the following fields according to the numbering in the images.

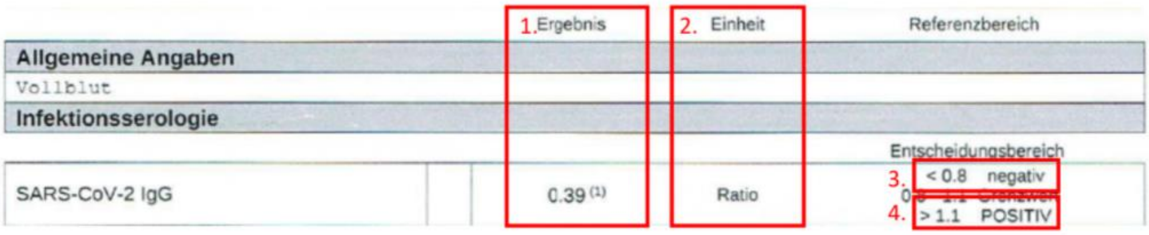

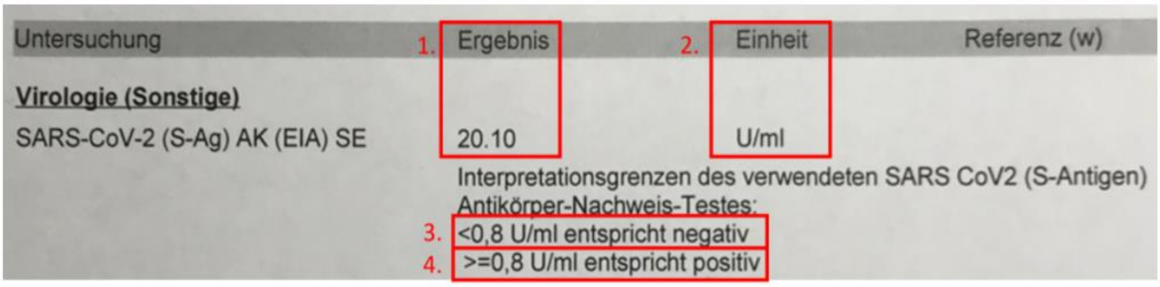

1. Result of the antibody test: ______
2. Unit: ______
3. Decision area negative: < ______
4. Decision area positive: ≥ ______

Have you had an antibody test **since the 2nd vaccination until the 1st booster vaccination** (**for basic immunization with two vaccinations**)?

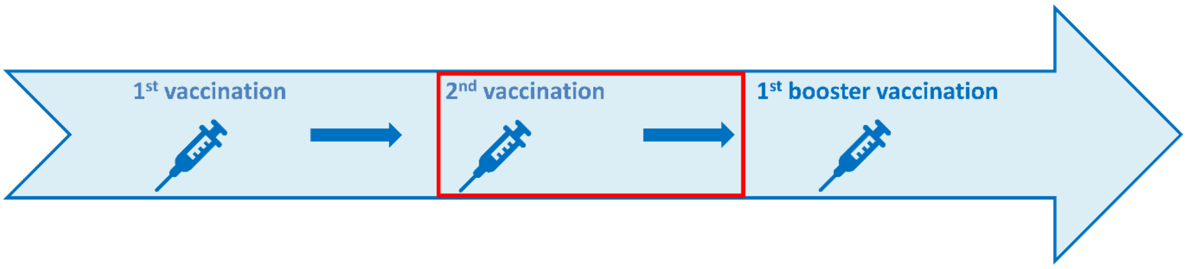

☐ Yes, antibodies positive

☐ Yes, antibodies negative

☐ No

Date of the (last) antibody test (DD.MM.YYYY): ________

Below you will find two examples of antibody test results. Please fill in the following fields according to the numbering in the images.

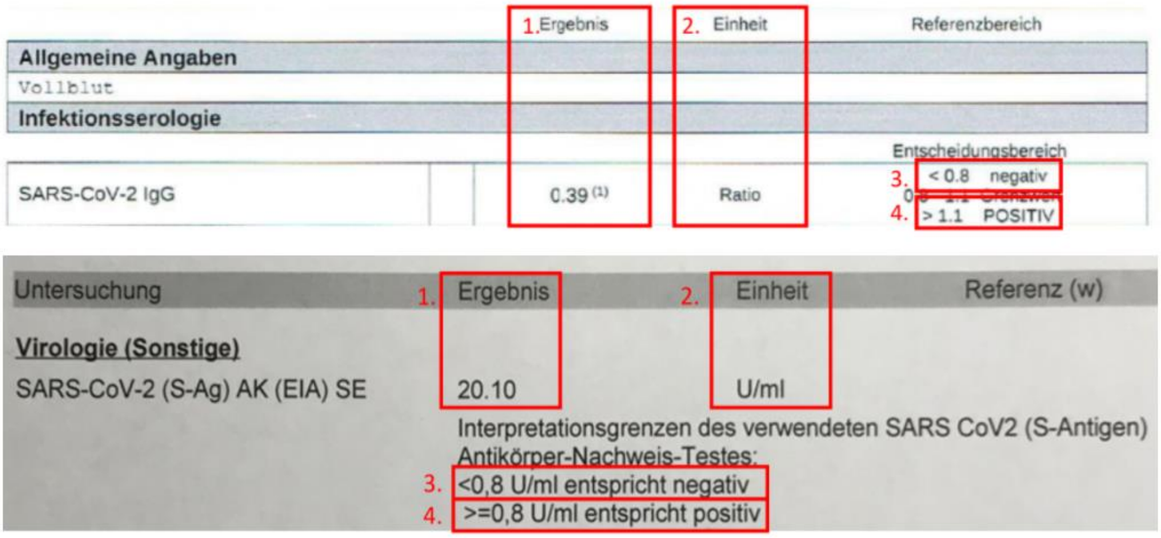

1. Result of the antibody test: ______
2. Unit: ______
3. Decision area negative: < ______
4. Decision area positive: ≥ ______

Have you had an antibody test **since the 1st vaccination until the 1st booster vaccination** (**for basic immunization with one vaccination**)?

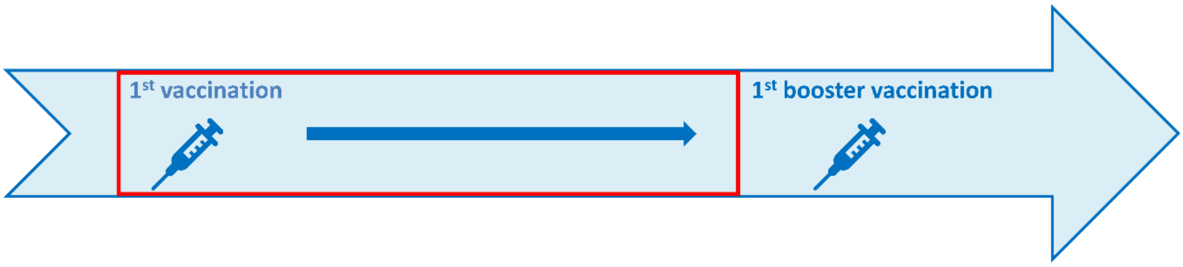

☐ Yes, antibodies positive

☐ Yes, antibodies negative

☐ No

Date of the (last) antibody test (DD.MM.YYYY): ________

Below you will find two examples of antibody test results. Please fill in the following fields according to the numbering in the images.

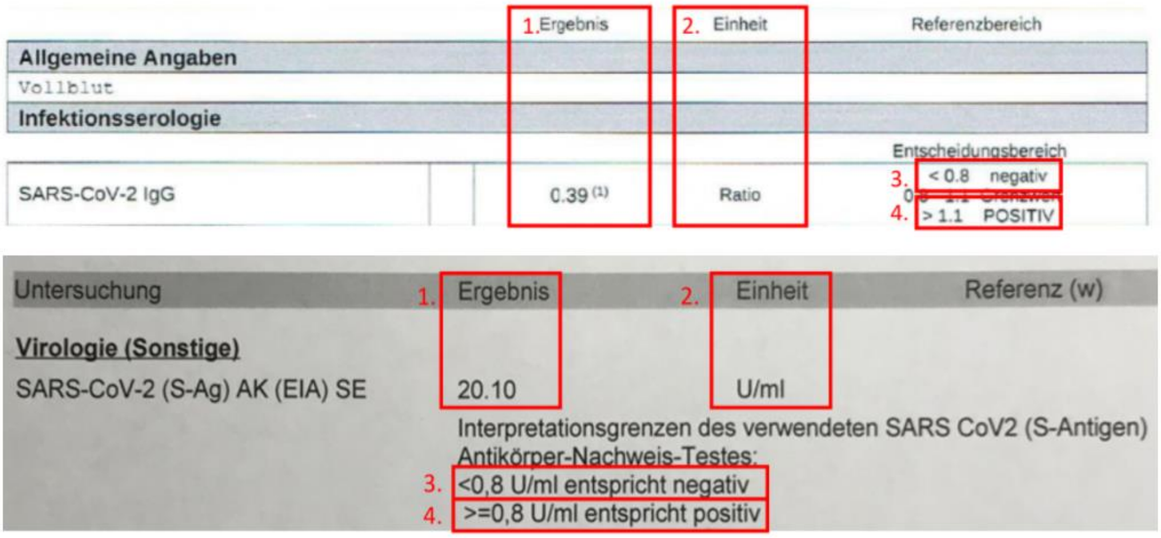

1. Result of the antibody test: ______
2. Unit: ______
3. Decision area negative: < ______
4. Decision area positive: ≥ ______

Have you had an antibody test **<u>since the 1st booster vaccination</u>** (**if booster vaccination received**)?

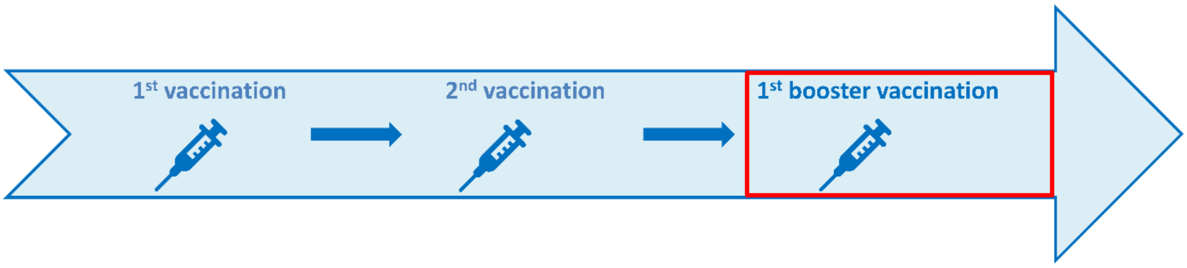

☐ Yes, antibodies positive

☐ Yes, antibodies negative

☐ No

Date of the (last) antibody test (DD.MM.YYYY): ________

Below you will find two examples of antibody test results. Please fill in the following fields according to the numbering in the images.

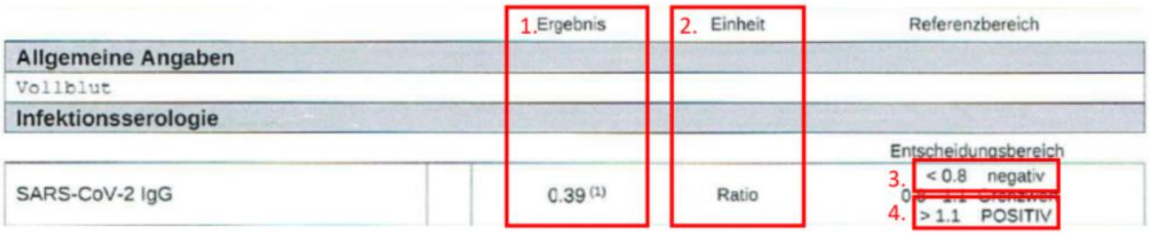

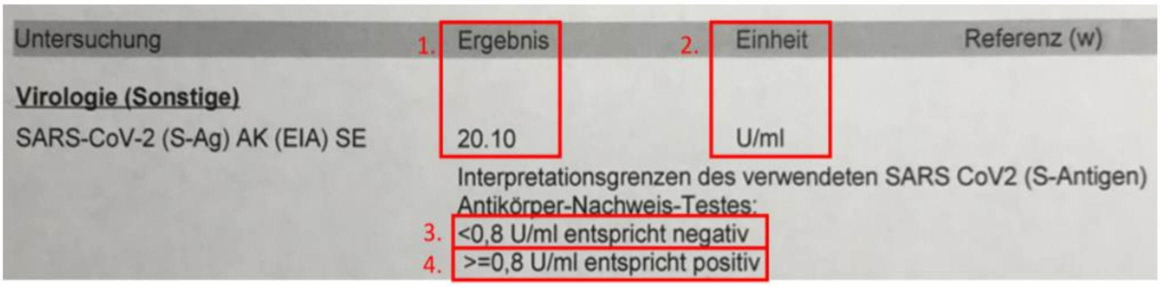

1. Result of the antibody test: ______
2. Unit: ______
3. Decision area negative: < ______
4. Decision area positive: ≥ ______

ELISPOT-Test:

Have you had an ELISPOT test **<u>since the 1st until the 2nd vaccination</u>** (**for basic immunization with two vaccinations**)?

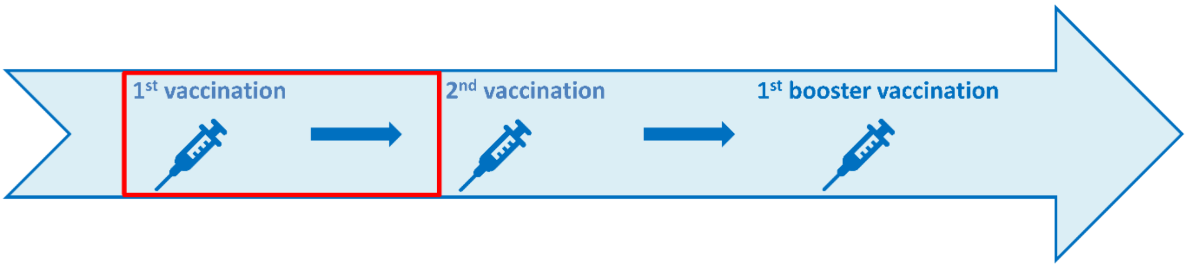

☐ Yes, ELISPOT positive

☐ Yes, ELISPOT negative

☐ No

Date of the (last) ELISPOT test (DD.MM.YYYY): ________

Have you had an ELISPOT test **<u>since the 2nd vaccination until the 1st booster vaccination</u>** (**for basic immunization with two vaccinations**)?

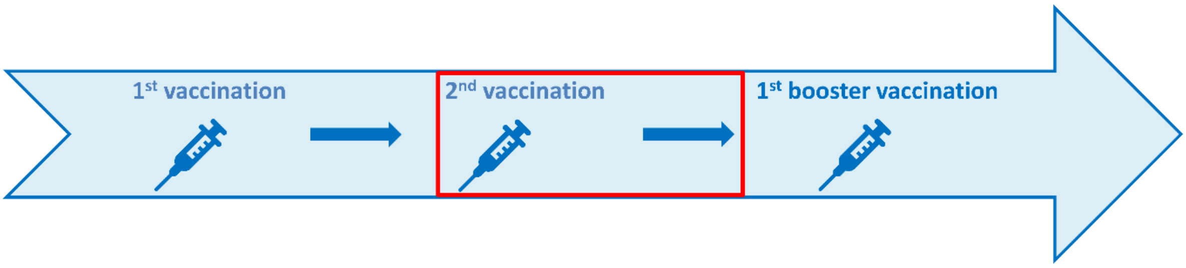

☐ Yes, ELISPOT positive

☐ Yes, ELISPOT negative

☐ No

Date of the (last) ELISPOT test (DD.MM.YYYY): ________

Have you had an ELISPOT test **<u>since the 1st vaccination until the 1st booster vaccination</u>** (**for basic immunization with one vaccination**)?

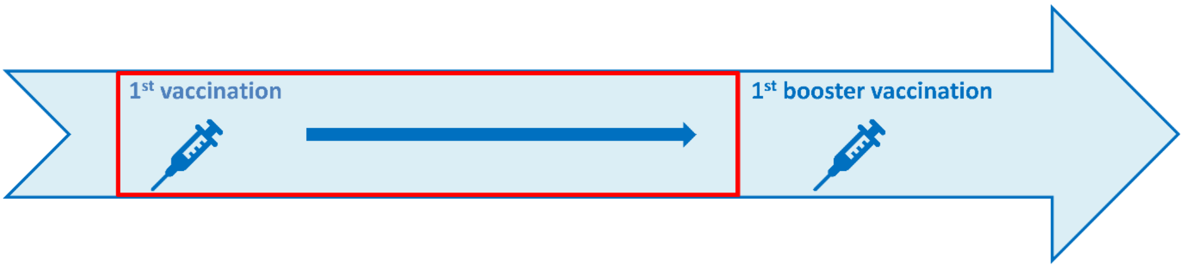

☐ Yes, ELISPOT positive

☐ Yes, ELISPOT negative

☐ No

Date of the (last) ELISPOT test (DD.MM.YYYY): ________

Have you had an ELISPOT test **<u>since the 1st booster vaccination</u>** (**if booster vaccination received**)?

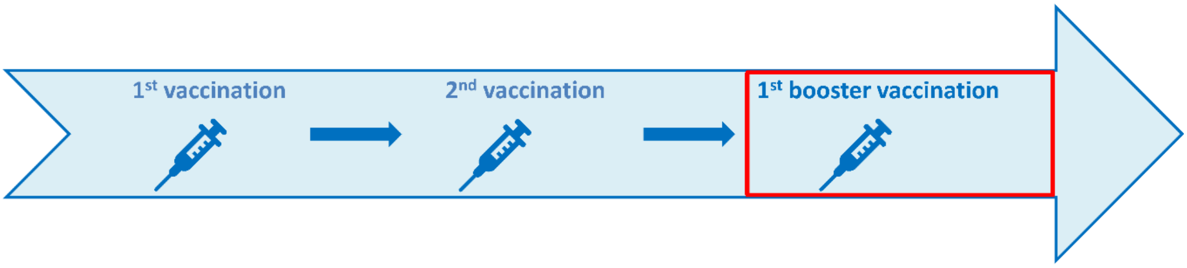

☐ Yes, ELISPOT positive

☐ Yes, ELISPOT negative

☐ No

Date of the (last) ELISPOT test (DD.MM.YYYY): ________

Patient-determined disease steps (PDDS)

Please read the choices below and choose the one that describes your own situation most appropriately.

This scale focuses mainly on how well you can **walk**.

You may not find a description that accurately reflects your condition, but please mark the category that describes your situation most closely.

Please choose one of the following answers:	
0 **Normal**<br>I may have some mild symptoms, mainly sensory due to my MS, but they do not limit my activity. When I have an episode, I return to normal as soon as the relapse is over.	☐
1 **Mild disability**<br>I have some noticeable symptoms due to my MS, but they are minor and have a small impact on my lifestyle.	☐
2 **Moderate disability**<br>I have no limitations in my ability to walk. However, I have significant problems due to MS that limit daily activities in other ways.	☐
3 **Walking impairment**<br>MS affects my activities, especially walking. I can work all day but sporting or physically demanding activities are more difficult than before. Normally I do not need a walking stick or other aids to walk, but I might need some help during a relapse.	☐
4 **Occasional use of a walking aid (walking stick use)**<br>I use a walking stick, a single crutch or some other form of support (e.g., touching a wall or leaning on someone's arm) to walk all or part of the time, especially when I walk outdoors. I think I can walk 8 metres in 20 seconds without a walking stick or crutch. I always need some help (walking stick or crutch) when I want to walk up to 300 metres.	☐
5 **Walking aid dependency**<br>To walk 8 metres, I need a walking stick, a crutch or someone to hold on to. I can move around the house or other buildings by holding onto furniture or touching the walls to support myself. I can use a scooter or wheelchair if I want to travel longer distances.	☐
6 **Bilateral support**<br>To walk up to 8 metres, I need two walking sticks or crutches or a rollator. For longer distances I can use a scooter or wheelchair.	☐
7 **Wheelchair**<br>My main form of mobility is a wheelchair. I may be able to stand and/or take a step or two, but I cannot walk 8 metres, even with crutches or a rollator.	☐
8 **Bedriddenness**<br>I cannot sit in a wheelchair for more than an hour.	☐
